# Systematic analysis of putative phage-phage interactions on minimum-sized phage cocktails

**DOI:** 10.1038/s41598-022-06422-1

**Published:** 2022-02-14

**Authors:** Felipe Molina, Manuel Menor-Flores, Lucía Fernández, Miguel A. Vega-Rodríguez, Pilar García

**Affiliations:** 1 Genética, Facultad de Ciencias, Universidad de Extremadura, Avda. de Elvas s/n, 06006 Badajoz, Spain; 2 Escuela Politécnica, Universidad de Extremadura, Avda. de la Universidad s/n, 10003 Cáceres, Spain; 3 Instituto de Productos Lácteos de Asturias (IPLA-CSIC), Paseo Río Linares s/n, 33300 Villaviciosa, Asturias Spain

**Keywords:** Antimicrobials, Bacteriophages, Phage biology, Microbiology, Applied microbiology

## Abstract

The application of bacteriophages as antibacterial agents has many benefits in the “post-antibiotic age”. To increase the number of successfully targeted bacterial strains, phage cocktails, instead of a single phage, are commonly formulated. Nevertheless, there is currently no consensus pipeline for phage cocktail development. Thus, although large cocktails increase the spectrum of activity, they could produce side effects such as the mobilization of virulence or antibiotic resistance genes. On the other hand, coinfection (simultaneous infection of one host cell by several phages) might reduce the potential for bacteria to evolve phage resistance, but some antagonistic interactions amongst phages might be detrimental for the outcome of phage cocktail application. With this in mind, we introduce here a new method, which considers the host range and each individual phage-host interaction, to design the phage mixtures that best suppress the target bacteria while minimizing the number of phages to restrict manufacturing costs. Additionally, putative phage-phage interactions in cocktails and phage-bacteria networks are compared as the understanding of the complex interactions amongst bacteriophages could be critical in the development of realistic phage therapy models in the future.

## Introduction

Bacteriophages, or phages, are the viruses of bacteria. They infect and replicate within their host and, subsequently, lyse and kill the bacterial cell to release new phage offspring. This “life” cycle confers phages with an intrinsic ability to act as antimicrobials, making them an interesting approach to overcome the current global antibiotic resistance crisis brought about by widespread antibiotic misuse. Indeed, the application of bacteriophages as therapeutics and biocontrol agents is increasingly gaining popularity, especially as a tool to fight against multiresistant pathogenic bacteria in several fields such as human health, agriculture, and the food industry ^1,2^.

Besides their lytic ability, another distinctive feature of phages is their high host specificity. This property would prevent disturbance of the surrounding microbiota when phages are used to remove pathogenic bacteria, which is a significant advantage over antibiotic therapy^3^. Nevertheless, in terms of efficiency, very specific phages (those infecting only a small number of strains) represent a challenge, making it necessary to use mixtures containing different phages with complementing host ranges, the so-called phage cocktails, to increase the number of target strains and become useful for a wider range of applications/patients^4^. Alternatively, a single phage with a broad host range or a polyvalent phage^5^ can be used to target multiple strains from a species or even multiple species in a community. However, polyvalent phages frequently show differences in infection efficiency (EOP) on different hosts due to, for instance, differential receptor-phage binding affinity.

From a logistical standpoint, therapeutic regimes involving only one phage would be simpler and potentially less costly. Indeed, phages that are part of a cocktail must be propagated individually, purified, and mixed prior to use, which might result in higher manufacturing costs. On the other hand, cocktails improve the commercial applicability of phage formulations partly due to their wider range of bacterial targets. Thanks to this ability, cocktails have the potential to be used without prior identification of the pathogens involved. Furthermore, phage cocktails may reduce or almost eliminate the chance of selecting phage-resistant bacteria^6^. To achieve this goal, phages in the cocktail should preferably belong to different families, so that strains becoming resistant to one phage can be potentially targeted by another phage in the mixture, as superinfection immunity is not possible between unrelated phages.

To date, there are no validated guidelines for the design of individual or mixed phage preparations targeting a given pathogen^7^. Therefore, in order to assemble a phage cocktail with optimal therapeutic potential, it is first necessary to identify candidate phages, and then determine their infection profiles against a diverse set of strains spanning the major lineages of the target bacterium. Nonetheless, bacteriophage cocktails should not be random mixtures of different bacterial viruses. For instance, Merabishvili et al.^8^ recommended some features to be considered for the selection of phages, which include being strictly virulent, not carrying virulence genes, and exhibiting high killing of the target pathogen, good stability and no transducing ability. In some cases, cocktail design would also involve the choice of bacteriophages with different bacterial cell wall receptor recognition sites^9,10^, thereby minimizing the emergence of resistance mutants. Additionally, phage candidates should exhibit the broadest host range possible against the target bacterium. When necessary, the host range might be broadened by serial passages on different bacterial strains, thus allowing adaptation of the bacteriophages by coevolution with their host^11^. Also, there are specific methods available to isolate phages with broad host range^12^ or to broaden the host range through site-directed mutagenesis of the host-range-determining regions (HRDRs) in the phage tail fiber protein^13^. Likewise, synthetic biology can be used to modify the phage host range by engineering phage genomes through modular swapping of phage tail components^14^.

In some cases, it is necessary to target several bacterial species with one formulation. This application would entail the design of even more complex cocktails, containing mixtures of bacteriophages active against each bacterial species. In this case, cocktail design is mainly based on the microbial composition and the frequency of occurrence of different bacterial species in the target infection site. Examples of complex cocktails include the commercial products Pyo- and Intestibacteriophage^15^.

Phage cocktail design must take into account not only interactions between phages and their hosts but also phage-phage interactions. The most well-known example of a phage-phage antagonistic relationship is the superinfection immunity mediated by temperate phages. Indeed, bacteria carrying a prophage are typically resistant to infection by related phages^16^. Moreover, phage satellites or pathogenicity islands can limit propagation of their helper phage, which confers some level of phage resistance to host bacteria^17^. Because of these and other interactions, the host range of a phage cocktail could be different from that predicted theoretically by combining the host range of each individual phage. Indeed, some phages might interact upon coinfection^18^ leading to synergetic or antagonistic effects^19^. For example, some cocktails show a narrower host range than the sum of the host ranges of all individual phages independently^20^. This could be due to host infection competition between phages, i.e., coinfection of the same host cell by multiple phages may lead to increased competition for limited cellular resources, thus reducing the fitness of an individual phage^21^. Antagonistic interactions might also occur when phages share the same receptor sites, or due to abortive infection mechanisms^22^. In turn, synergistic interactions between phages may be related to the rate of infection, the production of progeny, or the time between infection and progeny release^23^. More recently, a phage-phage communication system, arbitrium, that allows cross talk among close phages to regulate the lytic/lysogenic life cycles was described by Stokar-Avihail et al.^24^. Interestingly, this system may allow interactions with more genetically distant phages.

Overall, it seems clear that optimizing the number and combination of phages will be critical for designing effective therapeutic and biocontrol strategies. In a previous work^25^, the information provided by host range matrices was analyzed by building phage-bacteria infection networks (PBINs) and calculating the nestedness temperature (host range hierarchy of the phages). Subsequently, an estimator of phage cocktail size (Φ) was proposed considering some global properties of the host range matrices such as fill (fraction of successful infections), nestedness temperature, and number of bacteria. Here, we explore a new method, which considers the host range and each individual phage-host interaction, to design the phage mixtures that best suppress the target pathogens while minimizing the required number of phages.

## Results and discussion

### Designing phage cocktails from phage-bacteria infection networks (PBINs)

**Figure 1:**
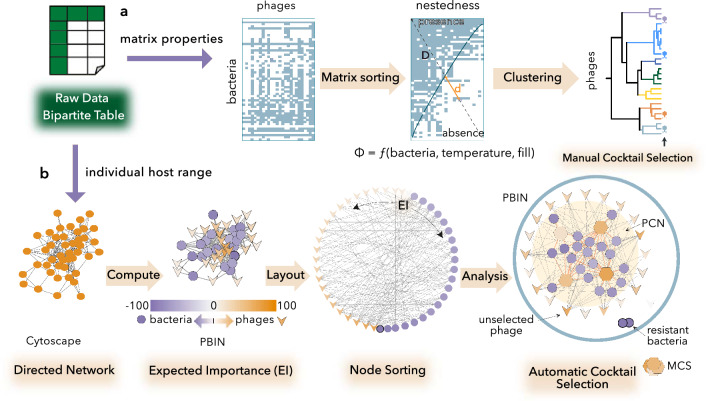
Alternative pipelines for designing phage cocktails. (**A**) Global properties of Phage Bacteria Infection Networks (PBINs). Nestedness algorithms reorder host range data and estimate the deviation (temperature) from a perfectly nested matrix by computing the relative distances (d/D) to the isocline of perfect order (blue line). The metric Φ considers global properties of the networks to estimate phage cocktail size^25^. Agglomerative hierarchical clustering is used prior to manual selection of the phages constituting each cocktail. (**B**) Automatic determination of the Minimum Cocktail Size (MCS). Bipartite phage-bacteria interaction matrices are imported into Cytoscape as directed networks, and the expected importance (EI) is measured for each node (see Methods). Nodes are colored and sorted by their EI and cocktails are designed, both heuristically and exhaustively, using the app PhageCocktail. The subnetwork (Phage Cocktail Network) harboring all susceptible bacterial strains and phages corresponding to the MCS is selected for each PBIN.

In a recent work^25^, a pipeline for designing phage cocktails based on global properties (fill, temperature, and number of bacteria) of the PBINs was proposed. However, host range matrices needed to be processed by two different sorting algorithms (BinMatNest and Ward) and the phage cocktail was not automatically generated (Fig. 1a). Here, we have developed a new approach (see Methods) that considers individually the host range of every phage, designs cocktails using only one sorting algorithm and automatically provides the Minimum Cocktail Size (MCS) (Fig. 1b).

A total of 50 datasets that include bacteria and phages from different sources, such as seafood, plants, livestock, dairy, sewage, clinical isolates and laboratory collection strains (Table 1) were used to build the PBINs and calculate the Expected Importance (EI) of every bacterium and phage (see Methods) in order to design the corresponding phage cocktails. Whereas most datasets (42 out of 50) comprised a single bacterial species, matrices harboring 2, 5 and 15 species were also surveyed (Table 1). The most frequent species was *Escherichia coli*, which appeared on 18 matrices, followed by *Pseudomonas aeruginosa* and *Staphylococcus aureus*, which appeared on four and three datasets, respectively. However, most bacterial species (43 out of 52) were present in a single dataset. In total, 2,877 bacterial strains, 899 phages and 52,688 phage-host interactions were evaluated. Subsequent processing of the PBINs led to the construction of subnetworks named Phage Cocktail Networks (PCNs), harboring all bacterial strains susceptible to phage infection as well as the phages contained in a hypothetical cocktail (Fig. 1b). Therefore, a processed network displays two types of source nodes (unselected vs. cocktail phages), two types of target nodes (resistant vs. susceptible bacteria) and two types of edges (unselected phages-bacteria vs. cocktail phages-bacteria interactions). Selection of the phages constituting the candidate cocktails was performed by applying both heuristic (Network Metrics) and exhaustive (Exhaustive Search) algorithms from the Cytoscape application PhageCocktail^26^ (freely available at https://apps.cytoscape.org/apps/phagecocktail), seeking the PCN that maximized the expected number of lysed bacteria while keeping a MCS. The heuristic algorithm employs EI values and considers phages with a broad host range (high EIp values) as better candidates for the cocktail, and bacteria susceptible to be infected by fewer phages (low EI_b_ values) as more problematic. Contrastingly, the exhaustive algorithm evaluates all the possible phage combinations, which warrants the identification of the smallest theoretical cocktail. However, this algorithm imposes a computational runtime penalty and may take several hours to execute^26^ when the number of phages is large. For instance, one matrix (named Korf et al. in Table 1) needed to be processed overnight. It must be highlighted that, even though alternative algorithms, such as agglomerative hierarchical clustering of phages by their host range, are faster, the exhaustive algorithm employed in this work warrants the best results (Díaz-Galián et al., submitted), i.e., the MCS infecting the highest number of bacteria.

**Table 1:** Experimental host range matrices used to generate Phage-Bacteria Infection Networks.

References	Matrix size	Hosts		Phages	Source
Hong et al. (2013)^a^	21	*Salmonella gallinarum*	7	3	Sewage, laboratory
Shende et al. (2017)^a^	40	*Escherichia coli, Bacillus subtilis*	8	5	Manure, sewage water
Mizuno et al. (2020)^b^	64	*Citrobacter rodentium*	32	2	Sewage treatment plant
Liao et al. (2019)^a^	68	*Escherichia coli*	17	4	Non-fecal compost, laboratory
Krasowska et al. (2015)^a^	76	*Bacillus subtilis*	19	4	Soil, laboratory
VHR1^b^, James L. Van Etten lab	78	*Chlorella variabilis*	6	13^c^	Laboratory
Gutiérrez et al. (2015)^b^	90	*Staphylococcus, Macrococcus caseolyticus*	45	2	Sewage treatment plant
Hwang et al. (2009)^a^	96	*Campylobacter jejuni*	16	6	Poultry, sewage, soil, laboratory
Kwiatek et al. (2015)^a^	100	*Pseudomonas aeruginosa*	20	5	Sewage, clinical
Hammerl et al. (2016)^a^	108	*Brucella inopinata*	36	3	Laboratory
Xie et al. (2016)^a^	120	*Salmonella enterica*	12	10	Manure, water, soil, laboratory, cattle feedlots
Magaré et al. (2017)^a^	125	*Bacillus novalis, Pseudomonas aeruginosa, Staphylococcus epidermidis, Bacillus cereus, Staphylococcus aureus*	5	25	Air
Álvarez et al. (2019)^a^	126	*Ralstonia solanacearum*	42	3	River water, potatoes, laboratory
Pereira et al. (2016)^a^	126	*Salmonella typhimurium*	42	3	Sewage, food, water, laboratory
Yu et al. (2016)^a^	155	*Pseudomonas syringae pv. actinidiae*	31	5	Soil, kiwifruit orchards
VHR5^b^, PMID: 22,936,928, 26,884,161, 10,430,569, 24,433,295, 22,834,906, 14,592,760	156	*Sulfolobus strains, Pyrobaculum arsenaticum,Pyrobaculum oguniense,Sulfolubus islandicus, Acidanus strains*	12	13	Acidic hot springs
Denou et al. (2009)^b^	156	*Escherichia coli*	26	6	Human feces
Schouler et al. (2021)^b^	168	*Escherichia coli*	56	3	Chicken fecal, recombinant phages
Gutierrez et al. (2010)^b^	195	*Staphylococcus epidermidis*	65	3	Women’s breast milk
Dias et al. (2013)^a^	200	*Staphylococcus aureus*	20	10	Livestock, sewage
Maura et al. (2012)^b^	219	*Escherichia coli*	73	3	Human feces
Galtier et al. (2017)^b^	219	*Escherichia coli strain*	73	3	Feces homogenates from murine gut samples
Alič et al. (2017)^a^	220	*Dickeya*	55	4	Orchid, wastewater
Molina et al. (2021) (3C)^a^	260	*Citrobacter freundii, Citrobacter youngae, Escherichia coli*, *Hafnia alvei, Klebsiella pneumoniae, Lactobacillus acidophilus, Lactobacillus casei, Lactococcus lactis ssp. Lactis, Salmonella typhimurium, Enterobacter aerogenes, Serratia marcescens, Shigella boydii, Shigella flexneri, Shigella sonnei, Yersinia enterocolitica*	26	10	Manure, sewage, laboratory, dairy
Salifu et al. (2013)^a^	270	*Rhodococcus equi*	27	10	Soil, equine
Arachchi et al. (2014)^a^	300	*Listeria monocytogenes*	50	6	Laboratory, seafood
Oh et al. (2017)^a^	324	*Bacillus cereus*	27	12	Laboratory, fermented food, soil
Wandro et al. (2019)^a^	330	*Enterococcus faecium*	15	22	Sewage human feces
Gunathilaka et al. (2017)^a^	348	*Escherichia coli*	12	29	Wastewater, laboratory
Jurczak-Kurek et al. (2016)^a^	360	*Escherichia coli, Pseudomonas aeruginosa, Salmonella entérica, Staphylococcus sciuri, Enterococcus faecalis*	60	6	Clinical, urban sewage
Litt and Jaroni (2017)^a^	378	*Escherichia coli*	54	7	Clinical, cattle feces
Romero-Suarez et al. (2012)^a^	416	*Xanthomonas arboricola pv. juglandis*	16	26	Walnut orchards
Wang et al. (2015)^a^	451	*Escherichia coli*	41	11	Cattle feces, human
Murphy et al. (2013)^a^	480	*Lactococcus lactis*	20	24	Dairy, Gouda-type cheese-producing plants Lactococcal phages
Sajben-Nagy et al. (2012)^a^	544	*Pseudomonas tolaasii*	34	16	Laboratory, mushroom
Sekulovic et al. (2014)^b^	555	*Clostridium difficile*	37	15	Animal and human fecal
Mangieri et al. (2020)^50^	630	*Escherichia coli*	30	21	Cattle and sheep feces, bedding material, sewage
Galtier et al. (2016)^b^	876	*Escherichia coli*	73	12	Sewage
Vu et al. (2019)^a^	1209	*Listeria*	31	39	Vegetable, seafood, sivestock, foods and food processing environments
VHR14^b^, Mathieu et al. (2020)	1344	*Escherichia coli*	84	16	Fecal samples of 1-year-old children
Molina et al. (2021) (3A)^a^	1456	*Escherichia coli*	56	26	Livestock feces, dairy, laboratory
Petsong et al. (2019)^a^	1692	*Salmonella enteritidis, Salmonella typhimurium*	47	36	Livestock
Jäckel et al. (2017)^a^	2147	*Ochrobactrum, Brucella inopinata*	113	19	Laboratory
Brady et al. (2017)^a^	2280	*Paenibacillus larvae*	40	57	Beehive
Lourenço et al. (2020)^b^	2744	*Escherichia coli*	98	28	Sewage water, laboratory
Fong et al. (2019)^51^	2806	*Salmonella enterica*	61	46	Sediment, cattle feces, sewage effluent, irrigation water, water tanks from an aquaculture facility
Gencay et al. (2019)^a^	2952	*Salmonella*	72	41	Laboratory, pork meat, environmental and wastewater samples
Korf et al. (2019)^a^	3200	*Escherichia coli*	64	50	Poultry, sewage, manure, clinical
Saussereau et al. (2014)^b^	8960	*Pseudomonas aeruginosa*	896	10	Cystic fibrosis isolates, laboratory
Mathieu et al. (2020)^a^	12,450	*Escherichia coli*	75	166	Fecal
Total	52,688		2877	899	

^a^Full reference available at Molina et al. (2021) https://doi.org/10.3389/fmicb.2021.564532.

^b^Downloaded from https://viralhostrangedb.pasteur.cloud/data-source/.

^c^The original matrix was trimmed to remove gaps.

**Figure 2:**
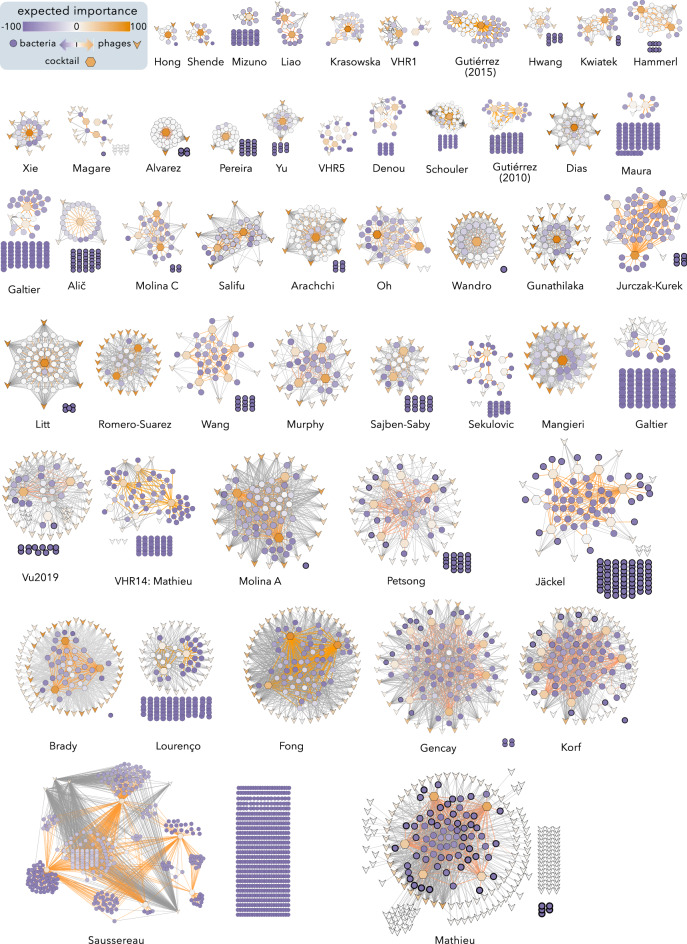
Graphical representation of Phage Bacteria Infection Networks (PBINs) and candidate Phage Cocktail Networks (PCNs) depicting the Minimum Cocktail Size (MCS). A total of 50 PBINs, each harboring a PCN subnetwork, were built and phage cocktails were designed using an exhaustive algorithm as detailed in Methods and sorted by increasing matrix size (Table 1). The shape of the nodes represents bacteria (), unselected phages () and cocktail phages (). The expected importance (EI) of each node represented by color shading so that more relevant nodes show more intense colors. Bacteria not susceptible to any phage (EI_b_ = -100) are clustered in grids. Lysis is indicated by dark (unselected phages) or orange (phage cocktail) lines.

Since Exhaustive Search outperforms Network Metrics, yielding smaller cocktails for the same number of infected bacteria (data not shown), we selected the PCNs obtained with the latter algorithm for further evaluation (Fig. 2). To compare both algorithms the expected importance (EI) of each node, determined by the Network Metrics algorithm (see Methods), is also shown. It is noteworthy that the Exhaustive Search preferably selected generalist phages displaying high EI values (Fig. 2). Although the current implementation of Exhaustive Search in PhageCocktail 1.1 is limited to combinations comprising up to 12 phages, this did not represent a problem in our search given that the size of the largest candidate phage cocktail predicted by the algorithm was 11.

**Figure 3:**
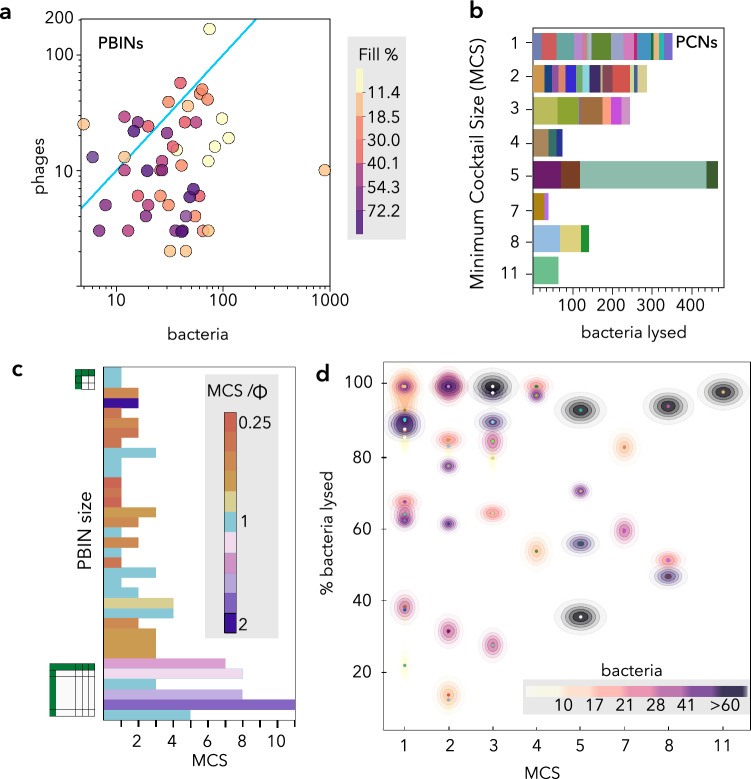
Characterization of networks (PBINs and PCNs) complexity and expected cocktail efficacy. (**A**) Analysis of PBINs complexity and symmetry. Each dot represents a single matrix (see Fig. 2) and the fill (%) is represented by color intensity. The cyan line indicates the position of symmetric matrices. (**B**) Distribution of PCNs grouped by the number of phages (MCS). Each bar sector represents a PCN and its length correlates with the number of bacteria lysed by the cocktail. (**C**) Comparison of MCS and Φ estimators. 34 PBINs, taken from Molina et al.^25^, were sorted by decreasing size. Bar length indicates the MCS, whereas color corresponds to the MCS/Φ ratio. (**D**) Expected cocktail efficacy vs. MCS. The fraction (%) of bacteria susceptible to at least one phage of the cocktail is shown for every PBIN. The number of bacteria is grouped by percentiles and represented by a density plot.

The complexity of a bipartite network depends on its size, symmetry, and fill (fraction of the maximum number of edges). For instance, square matrices result in more complex networks than similarly sized asymmetric networks because the maximum number of edges (possible phage-host interactions) increases. Additionally, the maximum algorithmic complexity^27^ of a PBIN is found for intermediate fill values because broad-host-range phages simplify cocktail design and any phage with an EI_p_ of 100 might be able to infect every host in a dataset. With this in mind, we sought to further characterize the PBINs by comparing the number of bacteria, phages and fill of each matrix (Fig. 3a). Most of the PBINs (80%) comprised more bacteria than phages, and the number of nodes (phages plus bacteria) ranged between 10 and 906. Large matrices were lightly filled suggesting a weak negative correlation between matrix size and fill (see below). Two outgroups harbored a significantly higher than average number of phages (166 vs. 18) and bacteria (896 vs. 58). These results showed the lack of consensus regarding the number of bacteria needed for host range determination, which coincides with the data obtained from a recent survey^28^ that found very little agreement in this regard between researchers (most answers stretched between 20 and 100 but one reached 800). Similarly, bias in data gathering, such as not considering narrow-host-range phages, might skew the fill of the PBINs.

### Analysis of minimum-sized phage cocktail networks (PCNs)

The distribution of the MCS in the different datasets (Fig. 3b) shows that most of the PCNs were indeed single phage formulations, followed by 2- and 3-phage cocktails, whereas larger (4, 5, 7, 8 and 11) cocktail sizes were less common. On the other hand, the number of bacteria in PCNs with a low MCS tended to be smaller. Thus, the minimum number of bacteria in large cocktails (containing four or more phages) was 10, whereas 19% of the PCNs corresponding to smaller cocktails contained nine or fewer bacterial strains.

In a previous work^25^, the size of candidate phage cocktails was estimated using a metric (Φ) that considers properties of PBINs such as nestedness temperature, fill and the number of target bacteria. However, Φ does neither evaluate individual phage-host interactions nor provide the minimum possible cocktail size. Comparison of both estimators, MCS and Φ, revealed (Fig. 3c) that Φ was larger than MCS for half (49%) of the PBINs, while being identical for most of the other half (37% of the total). Strikingly, Φ was smaller than MCS for the remaining PBINs (14%) most of which were fairly large networks. Remarkably, Φ takes into account the ecological and evolutionary information provided by the structure of PBINs, but it does not intend to achieve the minimum possible cocktail size. Also, Φ can be even larger than the number of phages assayed^25^ suggesting that additional phages should be isolated to perform effective biocontrol of the bacterial population. Conversely, the Exhaustive Search algorithm used to determine MCS examines individual phage-host interactions, thereby providing the optimal theoretical cocktail, rather than global properties of the network, and the expected success of the cocktails can be gauged by comparing PBINs and PCNs.

For fully successful cocktails, all the bacteria in a PBIN should also be part of the final PCN. With this in mind, the expected cocktail efficacy was estimated as the fraction (%) of bacteria susceptible to at least one phage in the cocktail by calculating the ratio of bacteria in every PCN/PBIN pair (Fig. 3d). These calculations revealed that 26% of the PCNs comprised all the bacterial strains from the original PBINs, which corresponds to the fraction (13 out of 50) of fully successful phage cocktails. While the fraction of bacteria lysed is generally high for all cocktail sizes (the median of all datasets is 74%), the variability of the expected efficacy decreases as MCS increases. This, however, is likely due to the asymmetric distribution of cocktail sizes (Fig. 3b). Interestingly, large phage cocktails did not exhibit low efficacy values (Fig. 3d) evincing that the algorithms used to design the cocktails do not spuriously increase the MCS. In the case of the heuristic algorithm, MCS is determined by favoring generalist phages with broad host range over specialists with narrow host range. In this regard, several experiments have shown that host range is a highly evolvable trait that relies on diversity, density and quality of hosts^29^. However, although host range expansion seems advantageous for phages, generalism may come with some tradeoffs such as a lower propagation rate^30^. Taking this into consideration, it would be interesting to develop alternative strategies for designing phage cocktails that maximize phage productivity, for example by selecting phages with large burst sizes and/or short latent periods, rather than those that minimize cocktail size.

### Phage-phage interactions: PBINs vs. PCNs

**Figure 4:**
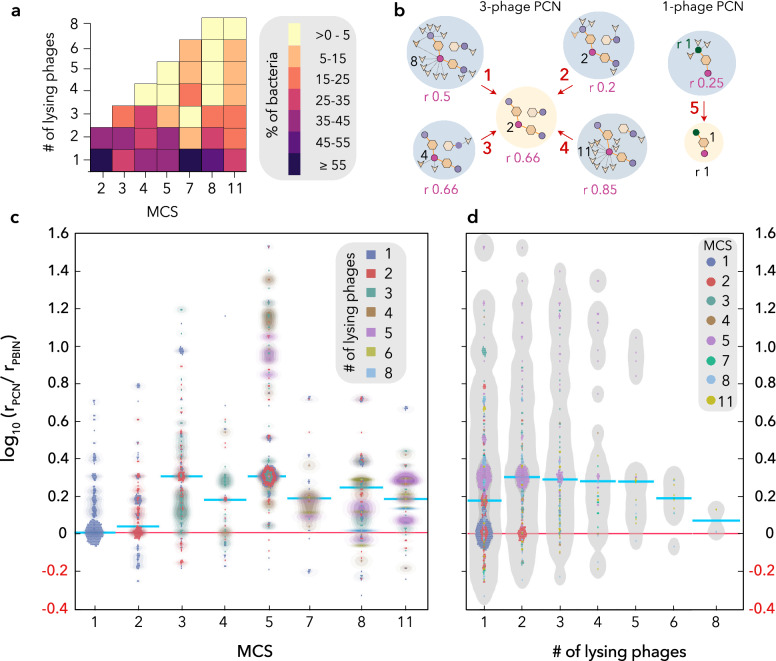
Analysis of phage-phage interactions: PBINs *vs.* PCNs. (**A**) Number of phages lysing bacterial strains for each cocktail size. The PCNs were grouped by MCS values and the fraction (%) of bacterial strains lysed by different number of phages is shown as a heatmap. (**B**) Examples of redundancy variations. Original PBINs and resulting PCNs are represented by blue and yellow shaded circles, respectively. Redundancy (r), the fraction of phages infecting a bacterial strain, is shown for one specific strain (red circle) in five PBINs and two PCNs. Additionally, the redundancy of a second strain is shown in PBIN #5. Examples of redundancy increase (1, 2, 5 ), constancy (3 ; 5 ) and decrease (4 ) are shown. (**C**) Redundancy variation (rv) of phage-host networks. PCNs were grouped by MCS and the redundancy change of the bacterial strains is represented as dots and density lines. The color indicates the number of lysing phages. The median of each distribution is shown by a cyan line. (**D**) Redundancy variation (rv) vs. number of lysing phages. Bacterial strains (dots) were grouped by the expected number of lysing phages and highest density region (HDR) box plots were generated. The color of the different dots indicates the MCS values and the cyan line represents the median of each distribution.

Although all the datasets evaluated in this work comprise experimental data, the actual performance *“*in vivo*”*of the phage cocktails designed here might be affected by phage-phage interactions that could hamper or facilitate coinfection (simultaneous infection of one host cell by several phages). To evaluate these putative interactions, PCNs were grouped by their MCS and then used to calculate the percentage of bacteria lysed by a given number of phages in all cocktails of the same size (Fig. 4a). This analysis revealed the intensity and frequency of phage-phage interactions for different cocktail sizes. Notably, the bulk of the bacterial hosts were lysed by a single phage, even in large cocktail networks, and only in the group comprising 3-phage cocktails there were more bacteria lysed by two phages than by a single one. Additionally, no bacterial host was lysed by 7, 9, 10 or 11 phages in the PCNs containing 7, 8 or 11 phages. These results suggest that, even though search algorithms preferably select generalist phages, the nestedness of the PCNs might be lower than that of the original PBINs^25^. Consequently, despite the fact that phages in nature span the continuum from specialist to generalist^31^, cocktail design might alter this equilibrium by favoring the selection of broad-host-range phages.

To further compare the structure of PBINs and PCNs, we studied the redundancy variation (rv) of phage coinfections. The redundancy of phage-host interactions (r), defined as the fraction of phages lysing a given bacterial strain in a network (see Methods), can increase, decrease or remain constant when comparing PBINs and PCNs (see examples in Fig. 4b). In turn, rv allows comparison of the redundancy found in a PCN compared to that of the original PBIN. This parameter ranges between a minimum (rv_min_), when only one phage in the cocktail infects the host, and a maximum (rv_max_), when all the phages lysing the host are considered. However, for single phage formulations rv = rv_min_ = rv_max_. Consequently, rv can never decrease (Fig. 4b) and its value depends on how many phages infect the host in the PBIN (see Methods). After grouping the PCNs by MCS the redundancy change (rv) of the different bacterial strains was determined as log_10_ (rPCN/rPBIN) (Fig. 4c). It can be perceived that the median values of rv increased (up to 2.5-fold) for all but single phage cocktails. This result implies that most bacterial strains were lysed by all the phages in the PBINs corresponding to single phage cocktails. For the rest of the networks there was a fraction of bacteria showing negative rv values while others exhibited an increase of up to 35 times (1.5 log). However, rv did not reach 0.8 in large (7, 8 and 11-phage) cocktails, i.e., redundancy increased less than 6.3 times. Concurrently, a paired samples t-test revealed (data not shown) a statistically significant 0.17 log difference between the means of rv and rv_min_ and a 0.27 log difference between rv and rv_max_, evincing the asymmetric effect of phage cocktails on rv. Remarkably, when rv values were grouped by the number of lysing phages instead of the MCS (Fig. 4d), the variability observed for rv decreased as the number of lysing phages increased. This result is partially explained by the low number of bacteria susceptible to a high number of phages in the cocktails, but it also reflects that these host strains are susceptible to be infected by a high number of phages both in the PBINs and PCNs. Conversely, bacteria lysed by a single phage in the cocktails were heterogeneous regarding their susceptibility to the other phages in their respective PBINs. Thus, some host strains susceptible to a single phage in the cocktails can be infected by multiple phages in the corresponding PBIN.

Recently, Niu et al.^19^ found different combinations of phages in cocktails displaying neutral, synergistic or antagonistic effects. Phages coinfecting the same host gain access to a common pool of proteins, which can be considered as intracellular public goods. Subsequently, a plethora of social behaviors may arise including cheaters, which do not contribute to the production of public goods, and cooperators, such as phages producing depolymerases that facilitate the adsorption of other phages to the host^29^. On the other hand, the phage-phage arms race might entail various strategies to prevent superinfection, such as triggering the premature lysis of bacterial cells infected by competitors^32^ or phage-encoded CRISPR-Cas systems^33^. Therefore, phage-phage interactions are worth examining carefully, as they might be detrimental or beneficial to the outcome of phage cocktail application. With this in mind, redundancy is a useful parameter to be considered in order to formulate cocktails with superior efficacy.

Nonetheless, the intensity of phage-phage interactions also depends, to some extent, on phage-host dynamics. For instance, the bacteriophage multiplicity of infection (MOI), ratio of phages to bacteria, is frequently employed to calculate the amount of phages that should be applied during dosing. Additionally, bacterial densities can change, and target bacteria may not be homogeneous regarding phage access^34^. As a result, redundancy variation analysis could be customized by incorporating additional information such as the MOI, the ratio of phageome to microbiome in the original environment, the burst size of different phages or their virulence index score^35^ to further improve its value during cocktail design.

### Size is not a determining factor for cocktail efficacy

**Figure 5:**
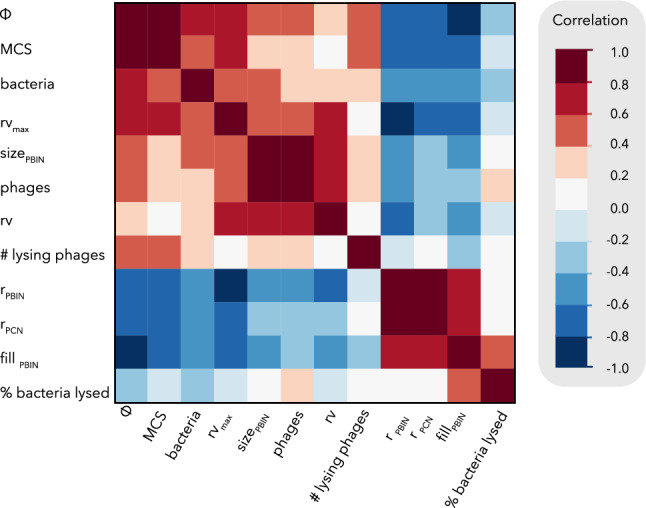
Correlation heatmap of different phage-host network parameters. Correlation values correspond to Spearman’s r. All strong positive and negative correlations were statistically significant (*P* value < 0.001).

Next, we assessed the potential correlations among the different parameters that characterize the networks by building a heatmap (Fig. 5). Interestingly, the expected success of the cocktails (% of bacteria lysed) displays a moderately positive correlation with the fill of the network, whereas its correlation with the number of phages, bacteria or cocktail size (MCS) is weak (< 0.4). A previous comparison of 31 datasets (Molina et al.^25^, data extracted from Chan et al.^36^) had already shown that the observed bacterial load reduction does not increase significantly with the size of the phage cocktail.

As shown in Fig. 3C, there is a strong positive correlation (Spearman’s r = 0.84) between MCS and Φ. Also, both parameters show a negative correlation with the fill of the PBIN, being particularly strong (> 0.8) in the case of Φ (Fig. 5). Additionally, MCS has a positive correlation with the number of target bacteria and rv_max_. In contrast, cocktail size is not correlated at all with the experimental rv (Fig. 4c), probably due to its negative correlation with redundancy in both PBINs and PCNs. These results indicate that datasets comprising more bacteria correspond to low filled networks, which leads to larger cocktail sizes and low redundancy values. To fully distinguish whether this reflects the different structure of small and large networks or some bias in data gathering by researchers, untrimmed datasets would be required.

### Concluding remarks

This study provides a simple and straightforward method to tackle cocktail design commencing with host range matrices. However, it must be noted that the use of binary networks, which only consider host range, leads to the loss of valuable information regarding infection efficacy^37^. It has been well established that a broad host range is not necessarily a synonym of success for bacteriophages^38^, especially because niche expansion may involve facing some antagonistic pleiotropic costs^39^, such as the loss of virulence^40^ and/or infection efficacy^41^. Moreover, the co-evolution of phages and their hosts often entails changes in bacterial community composition and structure^42^ or a decrease in the phage growth rate^43^. These strategies would ensure preservation of the host bacterial population for long-term exploitation, as well as increase extracellular stability of the newly-formed virions^44^. Likewise, the existence of synergy and/or antagonism among phages co-infecting the same host^19^ suggests that the development of new algorithms for phage cocktail design should involve the analysis of quantitative matrices and co-infection values. This method would allow the differentiation between simple short-term and more complex long-term biocontrol of host populations.

Immunity networks, built from CRISPR-Cas sequences, have been shown to influence the structure of complex PBINs due to their effect on virus diversification and host control^45^. Moreover, network proximity analyses have been used for rapid identification of potential drug combinations targeting 2019-nCoV/SARS-CoV-2^46^. Therefore, network-based methodologies can be applied not only to the control of microbes in foods, industrial settings and phage therapy applications, but also to study the dynamics of the microbiome, the evolution of host and viral populations^37^, and even to fight the SARS-CoV-2 pandemic^47^. Notwithstanding these examples of implementation of biological networks comprising viruses, the systematic analysis of PCNs is still far from being widespread. The present work attempts to tackle the potential offered by these strategies towards building a well-defined pipeline for phage cocktail design. However, further work will still be necessary to incorporate additional information regarding phage-phage interactions so that phage-bacteria networks accurately reflect the complexity of synergies and antagonisms amongst phages.

## Methods

### Data collection

A total of 50 datasets (Table 1) were gathered from three different sources: 34 tables were obtained from Molina et al.^25^, 14 were downloaded (https://viralhostrangedb.pasteur.cloud) from the Viral Host Range database^48^, and 2 were randomly selected after searching MEDLINE using the term “phage cocktail” and restricting the date from January 2019 onwards.

### Generation of phage-bacteria infection networks (PBINs)

The host range matrices (Table 1) were processed in the form of bipartite phage-host interaction matrices, reducing the lytic spectrum to either lytic or non-lytic interactions. These matrices were later transformed into directed networks (PBINs) by importing them into the Cytoscape platform^49^ using the plugin aMatReader. The Expected Importance (EI) of each phage (EI_p_) and bacterium (EI_b_) within the generated networks was calculated according to Eqs. 1 and 2, respectively:

1 $$EI_{p} = 100 \cdot \frac{{out_{d} }}{\left| B \right|},$$

2 $$EI_{b} = - 1 \cdot \left[ {100 \cdot \left( {1 - \frac{{in_{d} }}{\left| P \right|}} \right)} \right],$$

where | · | is the cardinality of the bacteria (B) and phages (P), and out_d_ and in_d_ refer to the outdegree and indegree of the nodes, that is, the number of arrows leaving source nodes (phages) or entering target nodes (bacteria), respectively. These equations consider that phages with a broad host range (higher outdegree values) are initially better candidates to constitute the cocktail, and that bacteria susceptible to be infected by fewer phages (lower indegree values) are more problematic for cocktail design. Additionally, it must be noted that the values generated by these two equations have opposite signs, which simplifies color labelling of source (phages) and target (bacteria) nodes in the networks (Fig. 1). An exception to this are phages that do not infect any bacterium or bacteria infected by every single phage in the dataset because both receive an EI of zero.

### Heuristic search of minimum sized phage cocktails

First, bacteria lysed by a single phage (EI_b_ = 1/ |P|) were identified for every network, and the corresponding phages were incorporated into the cocktail. Subsequently, the phage lysing the most bacteria (i.e., with the highest EI_p_ value) was included in the cocktail, unless no additional bacterial strains were lysed by the newly added phage. This process was iterated until all susceptible bacteria were predicted to be lysed by the cocktail, using the Network Metrics algorithm implemented in the Cytoscape app PhageCocktail 1.1.^26^.

### Exhaustive search of minimum sized phage cocktails

The Exhaustive Search option of the Cytoscape app PhageCocktail 1.1 was used to perform a combinatorial calculation of all the possible phage combinations from size 1up to a 12-phage cocktail size. Then, it returns the best phage combination (the one that lyses the most bacteria) for each size until reaching the maximum number of lysed bacteria. The last cocktail shown in the generated output file corresponds to the highest percentage of lysed bacteria, and, hence, was selected as the best candidate. The number of phages in this cocktail was then considered to be the Minimum Cocktail Size (MCS).

### Analysis of redundancy variation

Phage Cocktail Networks (PCNs) were built from the original PBINs by selecting susceptible bacteria and the phages constituting a given cocktail. Redundancy (r) was defined as the number of phages lysing a bacterial strain (indegree) relative to the number of phages in the network, and redundancy variation (rv) was measured for each bacterial strain by comparing the redundancy in the PCN (r_PCN_) to that in the whole PBIN (r_PBIN_) as indicated in Eq. 3.

3 $$rv = \log_{10} \left( {\frac{{r_{PCN} }}{{r_{PBIN} }}} \right) = \log_{10} \left( {\frac{{\frac{{in_{dc} }}{{\left| {MCS} \right|}}}}{{\frac{{in_{d} }}{\left| P \right|}}}} \right),$$

This metric varies between a minimum (rvmin), when only one phage in the cocktail infects a given bacterial host, and a maximum (rvmax), when every phage infecting each host strain in the PBINs is considered (Eq. 4).

4 $$rv_{\min } = \log_{10} \left( {\frac{\left| P \right|}{{\left| {MCS} \right| \cdot in_{d} }}} \right); rv_{\max } = \log_{10} \left( {\frac{\left| P \right|}{{\left| {MCS} \right|}}} \right),$$

## Data Availability

The authors confirm that the data supporting the findings of this study are available within the article.
